# Monitoring Cytogenetic Effects in Peripheral Blood Lymphocytes of Thyroid Cancer Patients Receiving Radioiodine Treatment

**DOI:** 10.3390/ijms27094049

**Published:** 2026-04-30

**Authors:** Igor K. Khvostunov, Elena Nasonova, Pavel Lobachevsky, Valeriy Krylov, Andrei Shurinov, Andrei Rodichev, Olga Korovchuk, Anna Geraskina, Ekaterina Shipilova, Petr Shegai, Andrei Kaprin

**Affiliations:** 1 Department of Radiation Biophysics, A. Tsyb Medical Radiological Research Center (MRRC)—Branch of the National Medical Research Radiological Center of the Ministry of Health of the Russian Federation (NMRRC), 10, Marshal Zhukov St., 249031 Obninsk, Kaluga Region, Russia; krylov@mrrc.obninsk.ru (V.K.); shurinov@mrrc.obninsk.ru (A.S.); rodichev@mrrc.obninsk.ru (A.R.); olga-korovchuk@mail.ru (O.K.); zanna@mail.ru (A.G.); 2 Laboratory of Radiation Biology, Joint Institute for Nuclear Research (JINR), 6 Joliot-Curie St., 141980 Dubna, Moscow Region, Russia; nasonova@jinr.ru (E.N.); lobachevsky@jinr.ru (P.L.); shipilova@jinr.ru (E.S.); 3 Department of Biophysics, Faculty of Natural Sciences and Engineering, Dubna State University (DSU), Universitetskaya St. 19, 141980 Dubna, Moscow Region, Russia; 4 National Medical Research Radiological Center of the Ministry of Health of the Russian Federation (NMRRC), 4, Korolev St., 249036 Obninsk, Kaluga Region, Russia; dr.shegay@mail.ru (P.S.); kaprin@mail.ru (A.K.); 5 Medical Institute, Peoples’ Friendship University of Russia (RUDN University), 6, Miklukho-Maklaya St., 117198 Moscow, Russia

**Keywords:** iodine-131, radioiodine therapy, thyroid cancer, adverse effect, blood lymphocyte dose, chromosome aberrations, biological dosimetry, multiplex fluorescence in situ hybridization

## Abstract

This study investigated cytogenetic damage in peripheral blood lymphocytes of 10 differentiated thyroid cancer patients who received multiple ^131^I radioiodine (RAI) treatments following total thyroidectomy. Blood samples were collected before the RAI therapy course and 2–3 days after the course for a few selected courses (from 1 to 3) for each patient. The cumulative average number of chromosome aberrations (CAs) per cell and its increment due to a selected RAI course were evaluated using the multiplex fluorescence in situ hybridization method (mFISH). An increase in the number of CAs was observed with the accumulation of RAI activity. The yield of these CAs per unit of accumulated RAI activity was, however, approximately three-fold lower than the respective yield for the incremented number of CAs in a selected course, thus demonstrating the elimination of CAs and/or aberrant cells with time. Biological dosimetry was performed based on the number of all types of CAs and in vitro mFISH calibration curves. With an average administered RAI activity of 3.40 ± 0.39 GBq, the average absorbed blood dose was 0.61 Gy (0.31–0.89:95% CI). Our results demonstrate that one-time administration of such activities of RAI was safe, since the commonly accepted threshold of 2 Gy for the blood dose was not exceeded.

## 1. Introduction

Radioactive iodine (RAI) therapy using ^131^I for patients diagnosed with differentiated thyroid cancer (DTC) is performed for the remnant ablation following total thyroidectomy and for the treatment of iodine-avid metastases. Although RAI therapy has been successfully used for more than 75 years, some adverse effects resulting from radiation exposure of normal tissues and organs remain a problem [1,2]. Consequently, the specificity of RAI metabolism, its biophysical properties, and the risk-to-benefit ratio of RAI therapy are still the subject of ongoing investigations [3,4]. The long-term irreversible adverse effects of RAI ablation, including secondary primary malignancy (SPM), xerostomia, and infertility, are of particular concern [4,5]. This requires a reliable personalized assessment of the absorbed dose from RAI and associated adverse effects to the patient’s healthy organs and tissues [6]. Unfortunately, up to now, there is no commonly accepted concept for RAI absorbed dose determination and prognosis of adverse effects due to the complex features of internal irradiation and insufficient available long-term follow-up data of DTC patient monitoring [4]. This especially concerns specific patients such as children and adolescents, patients treated with a considerable cumulative RAI higher than 22.2 GBq, patients who received additional treatment with stereotactic radiotherapy for suppression of isolated metastatic disease foci, patients with extremely rare diagnoses, and some others [7].

Although RAI therapy is considered a reasonably safe treatment, it has still been associated with an increased risk of SPM [2,8,9,10]. Based on the cohort study analysis of low-level exposure in childhood, a conclusion was made about a significantly increased leukemia risk [11]. In the case of repeated RAI treatment, it is important to ensure that the benefit of the therapy outweighs the potential risks due to higher individual and cumulative doses that increase the risk of various adverse effects [4].

Given the observation that no serious hematological adverse effects have been observed in patients who had received RAI absorbed dose of 2 Gy or less to the blood, which is the proxy for the bone marrow, this empirical cutoff is still in use as a guideline for nuclear medicine physicians [1,12]. No direct evidence of increased risk of SPM has been reported after a one-time RAI administration of 1.1–3.7 GBq [2]. However, the risk is clearly increased in patients who have been treated with a cumulative activity of RAI that is higher than 22.2 GBq [7].

For successful treatment of complicated patients, the specific planning strategy and personalization of RAI therapy are required [13,14]. To achieve personalization, a biological dosimetry can be effectively used for radiation dose assessment [15]. A number of approaches have been suggested for biological dosimetry based on the measurement of endpoints associated with the formation of radiation-induced double-strand breaks (DSBs) in cells [16,17]. DNA DSBs are considered one of the most critical radiation-induced DNA lesions that are responsible for cell death and other detrimental consequences of radiation exposure at the cellular level. Cells developed an efficient DSB repair system, and immunofluorescent detection of elements of this system, manifested microscopically as so-called radiation-induced foci, is exploited by some biodosimetry approaches. On the other hand, errors in the process of DSB repair by the non-homologous end joining pathway may lead to the formation of chromosome rearrangements that can be detected microscopically in mitotic cells as chromosome aberrations (CAs), and the number of CAs represents an effective endpoint for biodosimetry. The most widely used approach is a metaphase method of enumeration of various types of CAs, namely, dicentrics [18,19,20,21], translocations, dicentrics, and centric rings [22,23]. In general, biological dosimetry links the observed biological response to the calibration dose–response curve established in vitro [24]. The most frequently practiced methods exploit the linear-quadratic model for the description of dose–response and account for the dose rate effect in RAI treatment [21,22,23,25].

Analysis of CAs following RAI therapy allows for an estimation of a personal absorbed dose in peripheral blood lymphocytes (PBLs) for assessment of radiation risk and prognosis of late adverse effects. In addition, the persistently increased level of various types of chromosome damage is considered one of the primary precursors for the development of cancer [26].

The purpose of this study was to investigate the RAI-induced CAs using the most advanced, highly sensitive cytogenetic approach that exploits the multiplex fluorescence in situ hybridization method (mFISH), allowing identification of all individual chromosomes and rearrangements between them. The study involved cytogenetic examination of PBLs from 10 DTC patients subjected to multiple courses of RAI treatment during a long period, aiming to establish the effect of both the accumulated activity and one-time RAI exposure. The results obtained in the study were also used for the estimation of the absorbed dose to the patient’s blood on the basis of an in vitro established dose calibration curve for mFISH with the aim of evaluating the long-term risk of radiation adverse effects to normal tissues from RAI treatment.

## 2. Results

### 2.1. Features of RAI Treatment of the Study Patients

Blood levels of thyroglobulin (Tg), thyroid-stimulating hormone (TSH), and anti-thyroglobulin antibodies (anti-Tg Abs) are important indicators of a patient’s hormonal status that are considered for the appointment and evaluation of the success of RAI treatment. Tg is normally produced in differentiated thyroid cells and serves as a substrate for thyroid hormone synthesis. In patients with DTC, the blood level following total thyroidectomy and radioiodine ablation is considered a tumor marker. The RAI treatment data and blood levels of Tg, anti-Tg Abs, and TSH in patients involved in the study were measured for each course of RAI treatment and are summarized in Table 1 and Table 2.

**Figure 1:**
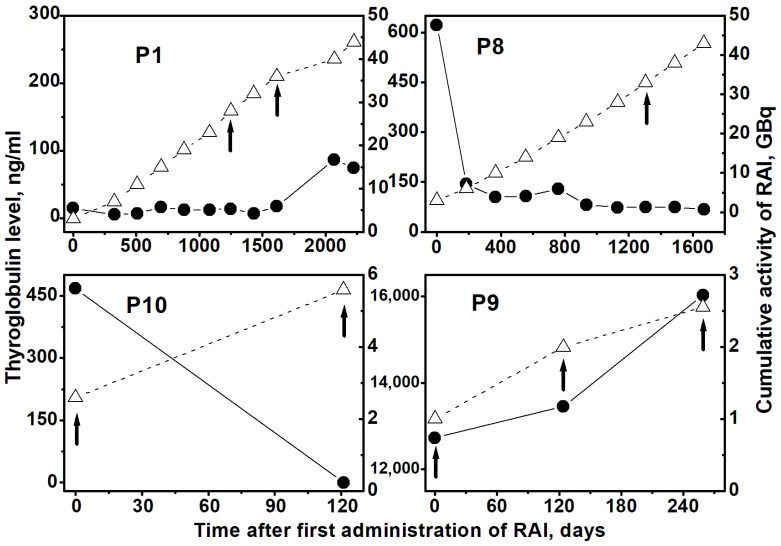
The dynamics of the level of thyroglobulin (solid circles, solid lines, left axis) and the accumulation of administered RAI activity (open triangles, dashed lines, right axis) for four examined patients in the process of RAI treatment, starting from the date of the first RAI administration. The vertical arrows mark the RAI courses accompanied by cytogenetic examination.

The dynamic of Tg level for a few selected patients (P1, P8, P9, and P10) is presented in Figure 1 along with accumulated activity. For patients P8 and P10, a decrease in Tg level is observed, which is usually expected following RAI therapy; however, this is not a case for patients P1 and P9.

### 2.2. Analysis of Stable and Unstable Aberrant Cells

**Figure 2:**
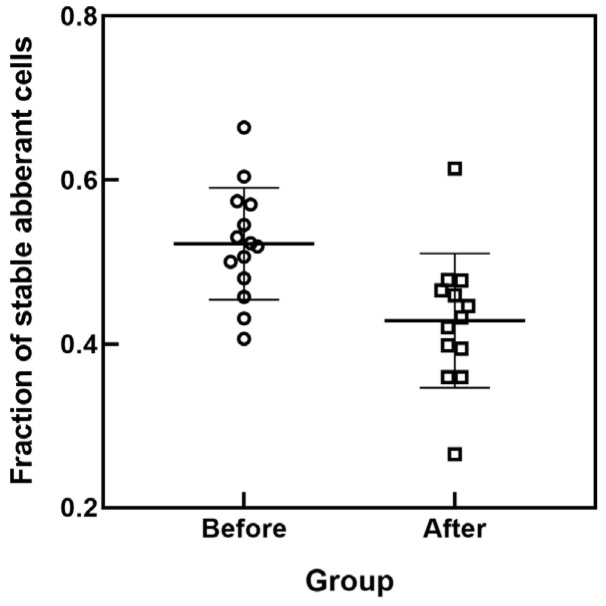
Scatter plots of the fraction of stable aberrant cells (in the population of aberrant cells) in samples from RAI patients collected before and after the RAI course.

The fractions of stable aberrant cells (in the population of all aberrant cells) in the examined patient’s samples collected before the RAI course are larger than those collected after the course, as evident from scatter plots presented in Figure 2. The difference between these two groups is statistically significant (*p* = 0.0033 for the unpaired *t*-test and *p* = 0.0076 for the paired *t*-test).

**Figure 3:**
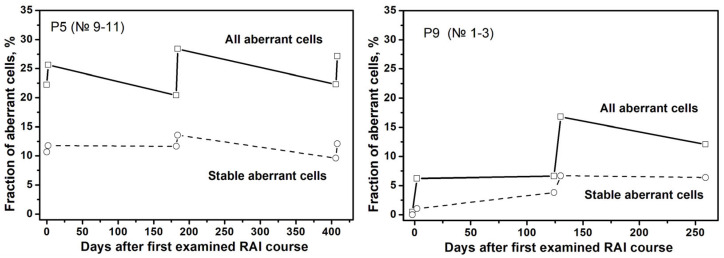
The fraction of all aberrant cells and stable aberrant cells in the two patients examined for three RAI courses.

The dynamic of changes in the fractions of stable and unstable aberrant cells in the progress of treatment is clearly demonstrated by the data obtained from a few selected patient samples (P5 and P9) that were collected for two or three different courses of RAI therapy. These data, expressed as fractions of stable and unstable aberrant cells in the population of all analyzed cells, are presented in Figure 3. The main observation is that the total number of aberrant cells increases following a one-time RAI course, and the major contributing factor for this phenomenon is an increase in the number of unstable aberrant cells. During the time interval before the next RAI course, the total number of aberrant cells decreases again, mainly due to the decrease in the number of unstable aberrant cells.

### 2.3. Analysis of the Total Number of Chromosome Aberrations

**Figure 4:**
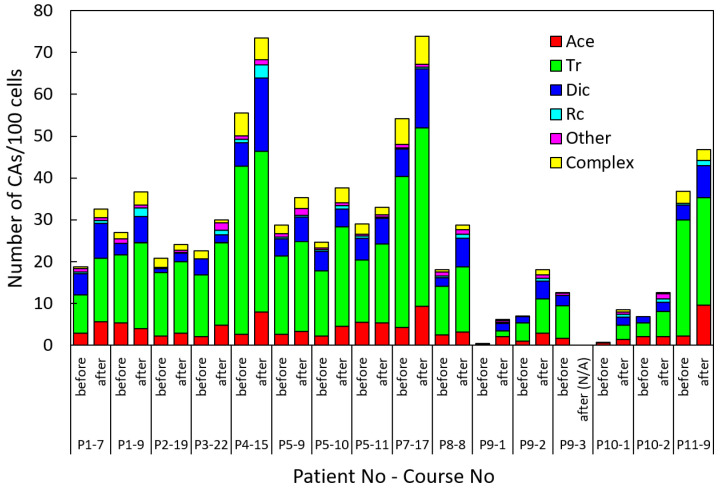
The number of CAs of different types and the spectra of observed CAs in examined patients before and after the RAI course. Ace—acentric fragments; Tr—all translocations; Dic—dicentrics; Rc—centric rings; other—all other aberrations; complex—complex aberrations.

The numbers of all types of observed CAs in examined patients before and after the given RAI course are summarized in Table 3 and presented graphically in Figure 4. The spontaneous level of CAs in PBLs of the healthy donors was 1.78 ± 0.31 CAs/100 cells (Table 4). On the other hand, before the first RAI course in two pediatric patients, the frequency of CAs was 0.47 ± 0.23 (P9-1) and 0.73 ± 0.33 (P10-1) or 0.58 ± 0.19 CAs/100 cells on average. Both of these values are significantly smaller than the spontaneous level in healthy donors (*p* < 0.05), consistent with the young age of pediatric patients. The major types of spontaneous CAs are also different between these groups. For healthy donors, the main contributions are acentrics and translocations (0.97 and 0.70 CAs/100 cells), while in two pediatric patients, these contributions are 0.45 and 0.06 CAs/100 cells, respectively (Table 3 and Table 4).

The maximum values before and after the RAI course were observed in the range of 54.2–73.9 CAs/100 cells in patients P4 and P7, resulting from 16 and 17 courses. Meanwhile, the minimum values were detected in the range of 0.73–18.1 CAs/100 cells in patients P9 and P10, as shown in Figure 4.

Based on the clinic personal medical records, the examined patients could be subdivided into two groups according to the RAI administration intervals: Group 1—P1, P5, P8; P9, P10, and P11 with ongoing RAI treatment, when an average of 3 months passed between consequent RAI courses, and Group 2—P2, P3, P4, and P7 with long-term therapy, when it was necessary to take breaks from 3 to 7 years between consequent RAI courses.

Six patients of the first group have been treated with a cumulative activity ranging from 2.55 to 46 GBq in two to eleven one-time RAI courses during 0.3–5.0 years. Four patients of the second group have been treated with the activity ranging from 60 to 80 GBq in 16 to 22 one-time RAI courses during 7.8–14.7 years. Thus, in Group 1, the cumulative activity during ongoing treatment did not exceed 46 GBq for a period of no more than 5 years (Table 1).

**Figure 5:**
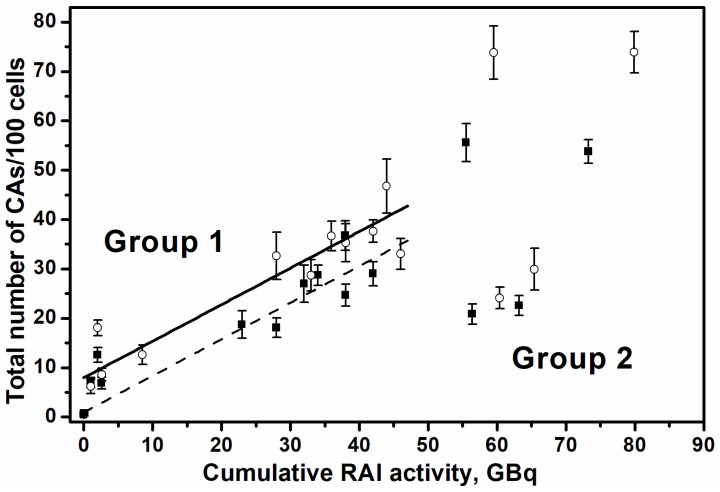
The total number of CAs before (closed symbols) and after (open symbols) the RAI course as a function of cumulative administered RAI activity. For regression analysis, Group 1 of patients was selected as described in the text. The dotted and solid lines show the results of linear regression for subgroups before and after RAI courses, respectively.

Figure 5 shows the total number of CAs (*N_CA_*) in the PBL of the patients as a function of cumulative prescribed RAI activity (*A*, GBq). The points represent the values before and after the RAI course. The lines show the corresponding linear relationships obtained following the regression analysis of the data for Group 1 of patients. The data were approximated by the linear regression $N_{CA}=N_{0}+kA$ assuming shared value for the parameter k, which reflects the yield of induction of CAs per unit of accumulated activity.

The following best-fit values were obtained for the parameters k = 0.74 ± 0.03 CAs/100 cells/GBq and for *N*_0_ = 0.98 ± 0.18 and 7.98 ± 1.48 CAs/100 cells for the “before” and “after” groups, respectively. The subtraction of $N_{0}$ values produces the average increment for $N_{CA}$ in a single RAI course as Δ$N_{CA}$ = 7.0 ± 1.66 CAs/100 cells. The average administered activity in a single RAI course for Group 1 of patients, calculated from the data in Table 2, equals <A_1_> = 3.35 ± 0.51 GBq. Therefore, the expected aberration induction rate is k_1_ = 2.09 ± 0.81 CAs/100 cells/GBq, and the ratio k_1_/k equals 2.82 ± 1.21. Thus, comparison of this value for k_1_ with the average yield of CAs per unit of cumulative activity, i.e., the value for parameter k, which is about 2.8-fold less, indicates that efficient elimination of CAs and aberrant cells occurs between consecutive courses of RAI treatment.

### 2.4. Analysis of the Spectrum of Chromosome Aberration

**Figure 6:**
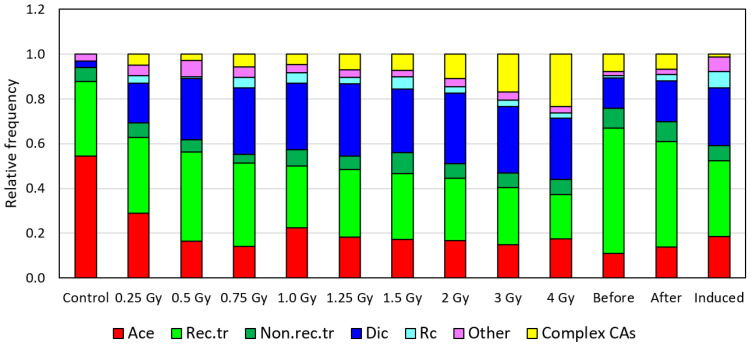
Normalized spectra of CAs in PBLs from donors following in vitro γ-irradiation at different doses (we used three male and three female donors) and in samples collected before and after RAI treatment of DTC patients. Data for patients represent average values for all samples collected before and samples collected after RAI therapy. Ace—acentric fragments; Rec.tr—reciprocal translocations; Non.rec.tr—nonreciprocal translocations; Dic—dicentrics; Rc—centric rings; other—all other aberrations; complex CA—complex aberrations.

**Figure 7:**
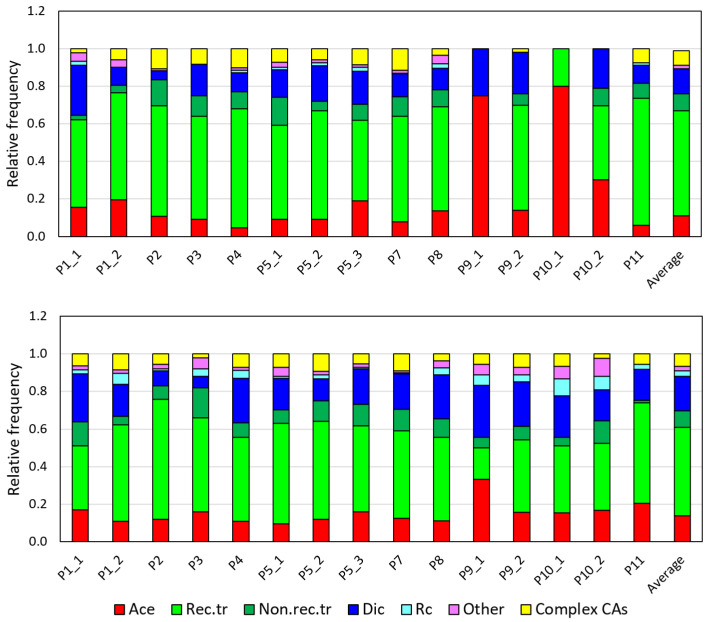
Normalized spectra of CAs in blood samples collected from study patients for cytogenetic examination before (top panel) and after (bottom panel) the one-time RAI course. Ace—acentric fragments; Rec.tr—reciprocal translocations; Non.rec.tr—nonreciprocal translocations; Dic—dicentrics; Rc—centric rings; other—all other aberrations; complex CA—complex aberrations.

Figure 6 and Figure 7 demonstrate spectra of CAs as relative (normalized to 1) frequencies of different types of CAs for easy visual comparison. We used contingency table analysis with the chi-squared test to compare the CA spectra expressed as the absolute (as required by the contingency tables method) measured number of CAs.

Analysis of the CA spectra in γ-irradiated donor cells indicated a statistically significant difference between background and radiation-induced spectra starting from 0.5 Gy (*p* < 0.0006). The low level of dicentrics and the lack of complex CAs in unirradiated cells account for this difference (Figure 6). No difference was detected between spectra in the range of 0.25–1.5 Gy (*p* = 0.30); however, a difference was statistically significant with the range extended to 2 Gy and more (*p* < 0.01). This difference follows from the increased contribution of complex CAs and the decreased contribution of translocations at large doses (Figure 6).

Analysis of CA spectra in cells from study patients shows that neither all spectra before RAI therapy nor after belong to the same statistical population (*p* < 0.0001). Such a result is not surprising given expected personal and treatment variability (Figure 7). However, when considering spectra before and after RAI therapy for individual patients (for which a few one-time courses were investigated), no significant difference was found between spectra before different courses of therapy for each of the patients P1 (*p* = 0.35) and P5 (*p* > 0.05), as well as between spectra after different courses of treatment for each of the patients P1 (*p* = 0.14), P5 (*p* = 0.42), P9 (*p* = 0.51), and P10 (*p* > 0.80).

This observation indicates the consistency of CA spectra for an individual patient obtained under similar conditions. We also compared spectra before RAI therapy in a range of patients with cytogenetic examination following a similar number of courses and accumulated activity (from 15 to 22 courses, activity from 51 to 76 GBq (patients P2, P3, P4, and P7)) and found no significant difference between these spectra (*p* = 0.22). However, the comparison of spectra after RAI therapy for these patients indicated a significant difference (*p* = 0.0002) that can be explained by substantially different one-time RAI activity in the investigated course (from 0.31 to 1.36 Gy).

Statistical analysis of one-time RAI-induced spectra using the contingency tables method is not possible since absolute values of the number of induced CAs of different types cannot be obtained directly. Instead, we calculated the frequencies per cell of one-time RAI-induced CAs by subtracting these frequencies before therapy from those after and then multiplying by the number of scored cells in the after-treatment group. Given, however, that negative values were obtained in some groups (due to limited statistics), which does not allow the use of the contingency table method, we compared only patients with non-negative values (P8, P9-1, P9-2, P10-1, and P10-2). Although substantial variations can be seen between spectra in this group due to limited statistics, no significant differences between CA spectra were found (*p* = 0.34).

In addition, the average across all patients’ spectra was calculated for the before- and after-treatment groups, and the difference between these groups was found to be statistically significant (*p* < 0.0001, using the real total number of aberrations of each type in each group as input data, Figure 6). This difference originates mainly from an increased fraction of unstable aberrations (dicentrics) and a decreased fraction of transmissible aberrations (translocations) in the after-treatment group as compared to the before-treatment group.

We also calculated the average spectrum of one-time RAI-induced aberrations (Figure 6). This spectrum was not significantly different from the spectrum of γ-induced CAs at 0.25 and 0.5 Gy doses (*p* = 0.065 and *p* = 0.165, respectively) and was different from spectra for all other doses (*p* < 0.047) (Figure 6). The major factor for this difference is the low fraction of complex aberrations in the RAI-induced CA spectrum that presumably can be explained by low-dose rate exposure during RAI therapy.

### 2.5. Complex Chromosome Aberrations

The data on the numbers of CAs of all types are summarized in Table 3 for in vitro irradiated PBLs from healthy donors and in Table 4 for PBLs of patients, including complex chromosome aberrations (CCAs) that involved three or more breaks in two or more chromosomes. In the control samples of healthy donors, as well as in the non-irradiated samples of pediatric patients (P9-1 and P10-1), CCAs were absent, as shown in Table 3 and Table 4. In all irradiated donor samples, CCAs were detected, the proportion of which in the total number of CAs increased from 4.8% at a dose of 0.25 Gy to 23.5% at a dose of 4 Gy (Table 4). In all examined patients, CCAs were detected in each of the studied blood samples, with the exception of two initial non-irradiated samples of pediatric patients (P9-1 and P10-1), as shown in Table 5. The maximum values of CCA frequency were observed in patients P4 (5.6–5.2 CCAs/100 cells) and P7 (6.2–6.7 CCAs/100 cells). The average frequency of CCAs for all studied blood samples was 2.08 ± 0.36 CCAs/100 cells.

**Figure 8:**
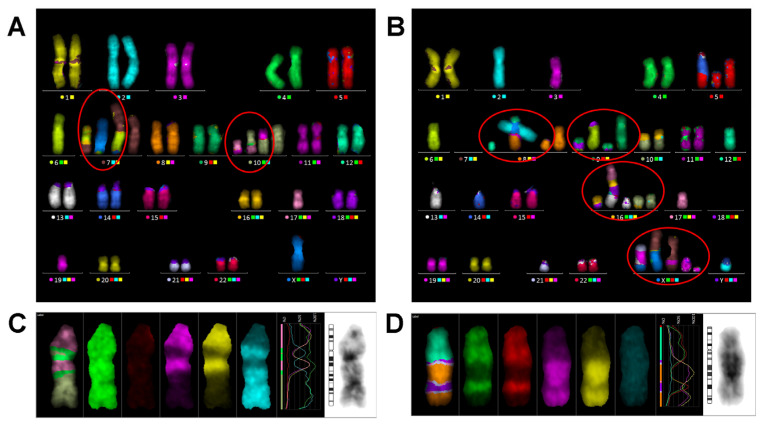
Representative karyotypes with complex aberrations revealed by the mFISH technique. Colors correspond to “pseudo colors” assigned by ISIS/mFISH 5.8 software. (**A**) Karyotype with two stable complex aberrations (shown in red circles) in P7-17 (before)—3/3/4 and 3/3/3; (**B**) Karyotype with two simple and four complex exchanges (shown in red circles) in P5-11 (after), containing 26 breaks in total in 20 chromosomes. All complex aberrations are unstable. (**C**,**D**) Pro-files of multi-translocated chromosomes: dic 10′-17′-10-17 in P7-17 (before) and dic 12′-8′-12-8 in P5-11 (after), respectively.

A few representative examples of karyotypes with different types of CCAs, classified as described in Methods, are presented in Figure 8.

### 2.6. Clonal Aberrations

For the patients with a long prehistory, all stable aberrations, particularly translocations, were checked for the presence of clonal aberrations. Translocations predominated in the group before RAI therapy, comprising from 50 to 75% of all detected CAs, while after in vitro ɣ-exposure, their frequency was 35–45% (Figure 6 and Figure 7).

**Figure 9:**
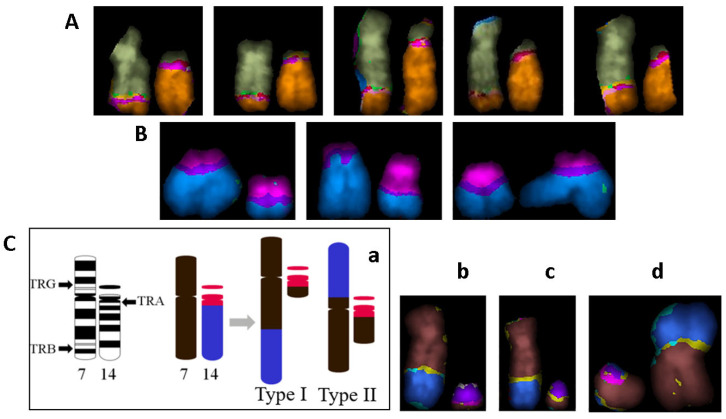
Clonal aberrations revealed by mFISH in examined patients. Colors correspond to “pseudo colors” assigned by ISIS/mFISH software. (**A**) Clonal translocation tr(8;10)(p1;q2) found in 16 cells of both P7 samples. (**B**) Clonal translocation tr(19;X)(q1;p1) found in three cells of P7. (**C**) (**a**) Schematic description of breakpoints involved in reciprocal translocation between chromosomes 7 and 14 (the arrows show the T-cell antigen receptor genes located at or near breakpoints). (**b**–**d**) tr (7;14) found in P7 and P1 (Type I) and healthy donor (Type II), respectively.

The vast majority of translocations are reciprocal. It is common practice to infer the clonal origin when aberrations are found in at least three cells [27,28]. However, according to the ISCN criterion [29], two cells with the same aberration are accepted as a clone. The examples of clonal aberrations in some of the examined patients are shown in Figure 9.

Defining three identical aberrations as clonal, we found four clones in three samples of patient P5, each clone consisting of three cells. Notably, in the last two samples collected 7 months later, none of these clones was found. Additionally, in sample 3, two cells containing identical complex CAs were detected: tr 1′-X + tr X′-1-X (Table 6).

In two samples, including P7, eight aberrant clones with one translocation were identified. The largest clone carrying tr(8;10)(p1;q2) was found to have a total of 16 cells in both samples, as well as three other clones: tr(3;22)(p2;q1), eight cells, tr(7;14)(q3;q1), four cells, and tr(5;6)(p1;q25), three cells. Another four clones were detectable only in one of two samples (Table 6, Figure 9). Several clonal translocations were also found in P1, 2, 8, and 11, including two clones consisting of two cells in P1 and P2 carrying two translocations (Table 6).

### 2.7. Biodosimetry

**Figure 10:**
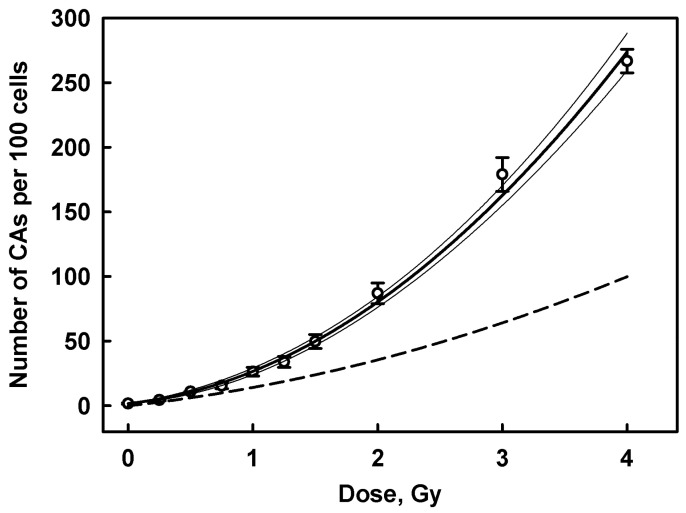
The dose–response for the number of all CAs induced in vitro by γ-irradiation of blood samples from healthy donors. Symbols represent the average number and standard error of CAs calculated across a range of two to seven measurements (donors). The bold solid line and thin lines show the result of non-linear regression and its 95% confidence band according to the linear–quadratic model (1). The dashed line is the calibration dose curve (2) obtained by taking into account the effect of the dose rate with $G\left(T\right)=0.251$, as calculated assuming an RAI course duration of 3 days.

Data for the CA dose–response used to establish a calibration curve are presented in Table 4. The dose–response and calibration curves are shown in Figure 10. The best fit values of parameters for the linear–quadratic model, Formula (1), are $\propto =10.5\pm 1.5\;{Gy}^{-1},\beta =14.4\pm 0.6\;{Gy}^{-2}$. The value for the intercept c=1.73 CA/100 cells was fixed at experimentally measured background number of CAs during the regression procedure, reflecting that the experimental value is more accurate than that obtained from the regression. These values of α and β parameters were used to calculate the calibration curve, Formula (2). To calculate the dose protraction factor $G\left(T\right)$, Formula (3), we accepted the effective ^131^I decay constant $\lambda =0.061\;h^{-1}$ [30] and the first half-time of repair τ=1.5 h [31,32].

The data and results for the calculation of absorbed doses to the patient’s PBL are presented in Table 7. The doses were calculated using Formula (7). The dose protraction factor $G\left(T\right)$ was calculated for each patient based on the value of T, the time interval between commencement of the RAI course and the patient’s blood sampling, and $∆Y\left(T\right)$ was calculated as an increment in the number of CAs in a one-time RAI course. The 95% CI and standard error of the mean for the absorbed doses were calculated according to IAEA recommendations [24]. Values for the initial dose rate R_0_ were calculated from its relationship with dose D (Formula (6)) and are presented in Table 7.

Comparison of the doses (Table 7) with activity administered in one-time RAI treatment course (Table 2) shows no correlation between these values (correlation coefficient R = 0.233, *p* = 0.4). The lack of correlation is also reflected in the values of the dose coefficient $k_{D}$ for individual patients in the dose–activity relationship $D=k_{D}A$ (where A is the activity administered in a one-time course). These values vary in the range 0.070–0.811 Gy/GBq, with an average value of 0.244 ± 0.049 Gy/GBq. Generally, the average absorbed dose to PBLs of study patients was 0.61 Gy (0.31–0.89: 95% CI) at an average one-time administered RAI activity of 3.40 ± 0.39 GBq.

## 3. Discussion

We examined CAs in PBLs from 10 DTC patients subjected to multiple courses of RAI therapy during a long period, aiming to estimate the absorbed dose to the patient’s blood on the basis of an in vitro established dose calibration curve for mFISH. Cytogenetic analysis in PBLs is widely used for epidemiological examination of individuals exposed to ionizing radiation, and the use of mFISH might be particularly reasonable and productive in cases of elevated exposures with increased complexity of cytogenetic damage [15,33]. Nevertheless, reports of cytogenetic examination of individuals exposed to ionizing radiation using mFISH are not very common, probably due to the high cost and time requirement of the method. These reports include mFISH analysis of long-term persistent chromosome damage following occupational exposure [34,35,36,37] and the Chornobyl accident [38]. Cytogenetic consequences of therapeutic exposures were also investigated using mFISH in a few studies [27,28,33,39,40]. Together with our last research on two DTC patients [23], the above references make an exhaustive list of published studies using mFISH.

The possibility to assess the fraction of stable aberrant cells by mFISH plays an important role, as they might be associated with an increased cancer risk. This study shows a minimal increase in the fraction of stable aberrant cells with accumulation of RAI activity and a decrease in the fraction of aberrant cells between two consecutive RAI courses to its level before the first course, as was also previously demonstrated in our study [23]. Therefore, the cancer risk of RAI treatment, evaluated by the cytogenetic effect of increasing the number of stable aberrations that can accumulate in the somatic cell population and potentially serve as a trigger for cancer [26], appears to be minimal.

Several patients were found to have clonal chromosome aberrations (Table 5), which are generally considered to be an indicator of long-standing damage to hematopoietic stem and/or progenitor cells. Clonal CAs arise from a single stem/progenitor cell that acquired the damage and passed it to the progeny. As CAs have to be multiplied during many cell divisions before they become visible in the peripheral blood as clones, the observed clones include CAs that have formed a long time ago and are not related to the recent treatments.

The presence of clones does not depend on treatment manner (P5 underwent EBRT with 46 Gy followed by eight RAI courses, while P7 had only 17 RAI courses) or on total CA frequency (for example, P4 and P7 had equally high CAs, but in P4, no clones were found). Apparently, the localization of treated metastases in close proximity to hematopoietic stem cell sites plays an important role.

Remarkably, translocations tr(7;14)(q3;q1) together with the other tr(7;14)(p1;q1) have previously been described by [41] using the G-banding technique and termed Type I and II (Figure 9C). These special translocations have been reported in PHA-stimulated lymphocytes of healthy donors, patients, and nuclear plant workers [41]. They are suggested to be T-cell-specific since the genes coding T-cell antigen receptors were shown to be located at or near breakpoints 14q11 and 7q34 ([28] and references therein). Two copies of both types of tr(7;14) were detected by means of the mFISH method in a patient undergoing radon spa therapy [28]. In total, we have detected tr(7;14) Type I in P7 (four cells), in P1 (three cells), one of the healthy donors (one cell), a pediatric patient’s sample before RAI (day 0, 2 cells) reported previously [23], and only one cell with Type II in a healthy donor now.

However, despite high RAI activity, no clonal CAs were revealed in the majority of examined patients. It is a good sign that stem/progenitor hematopoietic cells were not significantly affected by RAI therapy.

mFISH allows for the recognition and analysis of complex aberrations. As a result, with the exception of two pediatric patients, all others were found to have CCAs at initial examination (Table 7). The proportion of CCAs ranged from 2.2 to 11.5% of the total number of CAs, with a high proportion of unstable complexes. RAI therapy also induces a small number of CCAs in pediatric patients, primarily due to unstable CCAs. An increase in the number of CCAs was detected in the PBL of patient P1, while for all other patients, the number of CCAs decreased.

Thus, it can be concluded that RAI therapy does not cause a persistent accumulation of CCAs, particularly stable ones, or an increase in their complexity, as measured by the average number of chromosomes and breaks per complex (C/B) (Table 7).

The assessment of the absorbed doses to PBLs of examined patients based on the sum of all CAs revealed that a one-time administration of RAI was safe, as the dose threshold of 2 Gy was not exceeded, with a statistical confidence of 95% (Table 7). The lack of correlation between the absorbed dose and the one-time administered RAI activity and consequent wide range of individual dose coefficients (0.070–0.811) indicates that the details of activity distribution within each patient’s body and the individual rate of its excretion represent important factors determining the extent of cytogenetic damage and calculated absorbed dose. In the present study, we used the average effective ^131^I decay/clearance constant λ=0.061 h−1(T12=11.4 h) [30]. In the same study [30], the kinetics of RAI activity were investigated in a large group of RAI-treated patients (90 cases), and individual T12 values were found in the range from 8 to 27 h, thus pointing to a potential source of variability of individual dose coefficients and the lack of correlation between the administered activity and the absorbed dose.

Apart from the intra-study variability of dose coefficients, it is worthwhile to compare values from different studies. In our previous publication [22], we reported a similar value of dose coefficient $k_{D}=$ 0.238 ± 0.020 Gy/GBq (compared to 0.244 ± 0.049 Gy/GBq in this study) from the analysis of CAs in PBLs of DTC patients using the standard and FISH methods. Biodosimetry of RAI therapy in a group of 61 DTC patients based on the measurement of dicentrics was also reported in a recent study [21]. Similar to our study, no correlation was observed between the estimated absorbed dose to blood and the administered activity. However, the average value for the dose coefficient $k_{D}$ obtained in that study was 0.065 Gy/GBq, which is substantially lower than that obtained in our study. Such a discrepancy follows from a larger value for the factor $G\left(T\right)$ (0.455 compared to 0.25 in our study). In this study [21], the protraction factor $G\left(T\right)$ was calculated at 6 h, while blood samples were collected on days 3 and 7 after the RAI course; thus, the effect of damage repair and calculated doses were underestimated.

Another aspect of biodosimetry is related to the comparison of dose coefficients for different radiopharmaceuticals. Investigation of cytogenetic effects in patients treated with ^177^Lu-DOTA-TATE for metastatic neuroendocrine tumors [42] revealed somewhat higher levels of cytogenetic damage to PBLs and subsequently higher absorbed doses calculated by biodosimetry (1.04 Gy on average as estimated from data in [42]) as compared to RAI treatment (0.61 Gy on average, present study). However, given that larger activities of ^177^Lu-DOTA-TATE were administered per course (6.6 GBq on average), an estimation of the dose coefficient for this study produces a value of 0.16 Gy/GBq that is somewhat lower than for RAI treatment (0.244 Gy/GBq). Interestingly, an estimation of the dose coefficient for another radiopharmaceutical, ^177^Lu-PSMA-617, produces a somewhat higher value of 0.33 Gy/GBq [43]. Given that the physical properties of ^131^I and ^177^Lu are broadly similar (606 and 497 keV major β-emission), an interpretation of the observed differences would require detailed knowledge of biodistribution and excretion kinetics for each of the considered radiopharmaceuticals. For example, a relatively high mean effective half-time for ^177^Lu-PSMA-617 (41 h [44]) may account for a larger dose coefficient for this radiopharmaceutical compared to RAI. It is also important to note the substantial variability of the dose coefficients for ^177^Lu-DOTA-TATE (estimated from 0.06 to 0.21 Gy/GBq based on the data in [42]) that is in line with the variability of the excretion kinetics for this radiopharmaceutical, as reported in [45]. An estimation of the dose coefficient for a radiopharmaceutical with another β-emitting radionuclide, ^188^Re, produced a value of 0.22 Gy/GBq [46], which is similar to the RAI coefficient.

The lack of consistency in the biodosimetric estimation of the dose coefficient $k_{D}$ in different studies raises a question of the usefulness and reliability of this parameter and biodosimetry in general in monitoring effects of radionuclide therapy [6,15]. It is important, therefore, to consider a number of factors that significantly affect the results of biodosimetry. One of such factors is the assumption of a certain biological half-life time of radionuclide in the body that, in turn, depends on both the physical half-life time of radionuclide and the rate of the clearance of the radiopharmaceutical. These parameters depend on particular details of pathology and treatment; therefore, the comparison of the dose coefficient values obtained in different studies can be justified only for similar clinical data groups, the same type of diseases and treatment, and the same radiopharmaceutical.

Another important issue is the comparison of the biodosimetric dose estimates obtained using different endpoints, such as CAs (dicentrics or all types of CAs), micronuclei, γH2AX foci, etc. [15]. It is reasonable to expect that if an estimate is done correctly, i.e., it reflects the real physical dose, then the results should be consistent between different endpoints, and the lack of such consistency implies a methodological problem. We consider the biodosimetry, if applied correctly, as a convenient and trustworthy tool in monitoring the consequences of radionuclide therapy.

Blood levels of Tg are regarded as important markers measured in the course of RAI treatment. A reduction in Tg blood level to its normal value is considered one of the main indicators of successful RAI therapy. Two other indicators, such as anti-Tg Abs and TSH, are also important for prescribing RAI therapy. The normal level of Anti-Tg Abs is considered a standard indication for RAI therapy. At the same time, a radiologist prescribes RAI therapy, even with a high anti-Tg Ab level, if the Tg level is low. In addition, it is advisable to perform such a hormonal analysis in case of high TSH levels under the condition of thyroid hormone withdrawal. In particular, at low Tg and anti-Tg Ab levels, the indication for RAI therapy may be tumor invasion into skeletal muscles or metastases in the neck lymph nodes.

A decrease in Tg level after RAI treatment, followed by its low level, as demonstrated in Figure 1 for patients P8 and P10, is the typical example of Tg dynamics. Patient 10, a pediatric female aged 13, presents a quite unique case given her rare diagnosis—C73 (according to ICD) from the ovarian stroma of the right ovary. It is an extremely rare diagnosis when thyroid cancer emerges as a tumor from the struma ovarii with dissemination along the peritoneum. The quick positive response to RAI therapy, with Tg level decreasing from 450 ng/mL to virtually zero in one course, was observed, and only two courses of RAI therapy with a total administered activity of 5.6 GBq were required to achieve sustained remission. No significant features in terms of adverse cytogenetic damage were identified in this patient.

On the other hand, in some cases, RAI treatment does not lead to a successful outcome with a significant reduction in Tg levels, as demonstrated by examples in Figure 1 for patients P1 and P9. An increase in Tg level was observed in patient P1 during the last 10 and 11 courses, accompanied by a lack of RAI uptake in the patient’s body during diagnostic RAI administration. The case of patient P9, aged 6, is a typical example of refractory cancer that cannot be treated with RAI therapy. The initial extremely high Tg level of 12,730 ng/mL did not decrease after three courses of treatment with a total activity of 2.55 GBq but instead increased to 16,033 ng/mL. For this reason, RAI therapy was interrupted, and an alternative targeted therapy was recommended for this patient.

## 4. Materials and Methods

All procedures performed in studies involving human participants were in accordance with the ethical standards of the institutional and/or national research committee and with the Helsinki Declaration of 1975 (revised in 2013) and its later amendments or comparable ethical standards (Ethics Committee of A. Tsyb Medical Radiological Research Center—branch of the National Medical Research Radiological Center of the Ministry of Health of the Russian Federation (protocol No. 762 on 22 November 2022)).

### 4.1. Patients

A total of 10 patients, including 1 male and 9 females aged from 6.0 to 75.7 years and treated for DTC, were enrolled in the study. Individual patient data, such as age, sex, weight, diagnosis according to ICD, primary tumor details (histological type, presence of metastasis, and its location), and treatment-related information, including total administered RAI activity, number of RAI courses, length of therapy, and EBRT, are summarized in Table 1. All patients underwent total thyroidectomy. The DTC diagnosis C73 according to the ICD of various Tumor Node Metastasis stage was confirmed for all patients by postsurgical histopathology.

All patients underwent a number of ablative RAI therapy courses that varied from 2 to 22 (aqueous solution of Na^131^I, oral administration), with the cumulative activity ranging from 2.55 to 80 GBq. Some of the patients also received EBRT prior to the commencement of RAI therapy, as shown in Table 1 and Table 2. The dynamic of the level of thyroglobulin was followed up during all RAI courses. Thyroid hormone withdrawal was performed 3–4 weeks before RAI therapy for all patients.

### 4.2. Study Design and Blood Sample Collection

Peripheral blood samples for cytogenetic tests were collected from the patient’s median cubital vein before and after a few selected RAI courses. The time interval between “before” and “after” sampling corresponded to the period of the patient’s isolated stay (from 2 to 3 days). The number of cytogenetic tests per patient, including both “before” and “after” samples, varied from 2 to 6. For each blood sampling for the cytogenetic test, the following information was recorded: the sequential number of the selected RAI courses, one-time administered activity, accumulated activity, and the duration of treatment up to the commencement of the given course. This information is summarized in Table 2.

To establish a calibration curve for biodosimetry, blood samples were collected from 6 healthy donors (three males, three females, 26–40 years old, non-smokers) and exposed to a range of ^60^Co γ-rays with doses from 0.25 to 4 Gy on the ROCUS-M irradiator at a dose rate of 0.4–0.82 Gy/min, as shown in Table 4.

### 4.3. Blood Sample Processing and mFISH Chromosome Staining

Blood samples were processed according to the standard protocol described elsewhere [24]. In brief, 0.7 mL of blood was diluted in 8 mL of MEM supplemented with 20% FCS, 1% PHA, 1% L-glutamine, and 1% antibiotics (penicillin/streptomycin) and incubated for 48 h at 37 °C in a humidified atmosphere of 5% CO_2_. Metaphases were accumulated by the addition of colcemid to the culture to a final concentration of 0.2 µg/mL 2–3 h prior to the end of incubation, followed by hypotonic treatment (0.075 M KCl) and further fixation (methanol: acetic acid, 3:1). The cell suspension was dropped onto a wet slide, air-dried, and stored at −20 °C until mFISH staining. All slides were coded for blind analysis.

Slides were stained using a whole-genome painting 24XCyte mFISH probe kit (MetaSystems Hard & Software GmBH, Altlussheim, Germany) according to the manufacturer’s protocol. mFISH images were captured with a 63× oil immersion objective using a Zeiss Z2 Axio Imager (Carl Zeiss Microscopy GmBH, Jena, Germany). The chromosomes were identified and analyzed using ISIS/mFISH software (MetaSystems, Altlußheim, Germany), which assigns “pseudo colors” based on the unique combinations of fluorochromes enabling the identification of 22 pairs of autosomes, X and Y sex chromosomes, and all rearrangements between them.

We used the classical scoring system to record the observed CAs. All detectable aberrations were subdivided into simple breaks originating from one DSB, simple exchanges originating from two DSBs in two chromosomes, and CCAs that involved three or more breaks in two or more chromosomes.

Simple breaks included excess acentric fragments not associated with exchanges and truncated chromosomes (denoted hereafter as ace). Simple exchanges comprised reciprocal and nonreciprocal translocations (denoted as Rec.tr and Non.rec.tr, respectively), dicentrics (Dic), centric rings (Rc), and others (acentric rings, inversions, etc.), including complete, incomplete, and one-way forms, as described by [47,48]. Complex aberrations were described by the ratio C/A/B—each letter representing the number of involved chromosomes, arms, and breaks, respectively. The total number of breaks was calculated as a sum of simple breaks, simple exchanges multiplied by two, and B-values (from the C/A/B ratio) of all complex exchanges.

CAs were also classified according to their transmissibility to daughter cells. Reciprocal translocations (the complete form) were regarded as stable/transmissible; dicentrics, acentrics, and all nonreciprocal (incomplete and 1-way) forms, which may lead to the loss of genetic material and cell death, were regarded as unstable/non-transmissible. Complex aberrations were considered non-transmissible if they contained at least one non-transmissible part. The breakpoints of clonal aberrations were determined by means of ISIS 5.8 software containing a G-banded ideogram within the resolution limits of mFISH.

Based on the classification of CAs, analyzed cells were also classified into three categories: normal cells, i.e., cells without CAs, aberrant unstable cells, containing at least one unstable CA, and aberrant stable cells, containing only stable/transmissible aberrations.

Culturing of the patient’s cells and chromosome preparation were carried out at the MRRC. Samples of healthy donors were processed at JINR according to the same procedure. mFISH painting and cytogenetic analysis were performed at JINR.

### 4.4. Biodosimetry Approach

The CA dose–response calibration curve was approximated using the classical linear-quadratic model:

(1) $$Y\left(D\right)=c+\alpha D+\beta D^{2},$$

where $Y\left(D\right)$ is the number of observed CAs/100 cells following exposure at a radiation dose D and *c*, *α*, and *β* are the model parameters. For the dose–response of CAs induced by the RAI course, we used the expression that assumes correction for the dose rate effect introduced by the dose protraction factor $G\left(T\right)$ that accounts for the contribution of repair processes during irradiation with variable and relatively low dose rates as follows:

(2) $$∆Y\left(T\right)=\;\alpha D+G(T)\beta D^{2},$$

where *T* is the time interval between commencement of the RAI course and the patient’s blood sampling and fixation and $∆Y\left(T\right)$ is the increment in the CA number in the given RAI course. We calculated $G\left(T\right)$ using the generalized Lea–Catcheside formula [49,50]:

(3) $$G\left(T\right)=\frac{2}{D^{2}}\int_{0}^{T}R\left(t\right)dt\int_{0}^{t}\varphi (t-t^{\prime })R(t^{\prime })dt^{\prime },$$

where $R\left(t\right)$ is the dose rate as a function of time *t* and φ(t) is the repair kinetics and represents, in the context of the model, the probability that a sublethal primary lesion remains unrepaired at time *t* after its formation. For the calculation of φ(t), we used an incomplete repair model

(4) $$\varphi (t)=\frac{1}{1+\frac{t}{\tau }}\;,$$

where τ is the first half-time of repair [31,32].

The dose rate as a function of time can be approximated as a single exponential function, as suggested by [51,52]:

(5) $$R\left(t\right)=R_{0}e^{-\lambda t},$$

where *λ* is the effective decay coefficient determined by ^131^I body retention/excretion and radioactive decay and *R*_0_ is the initial dose rate, which is determined by the magnitude of the prescribed ^131^I activity and the physiological characteristics of the patient. In the framework of the used model, the relationship between the dose and the initial dose rate is described by the following equation:

(6) $$D=R_{0}\int_{0}^{T}e^{-\lambda t}dt=\;\;R_{0}\;\frac{\left(1-e^{-\lambda T}\right)}{\lambda },$$

reflecting that the integral of dose rate over time is the absorbed dose.

It follows from consideration of Equations (3) and (6) that the value of $G\left(T\right)$ does not depend on the value of $R_{0}$, meaning that the effect of the dose rate is determined not by its initial value but by the dynamics of the decrease in dose rate and repair kinetics. We also tested a two-exponential model for $R\left(t\right)$; however, we obtained similar results as with the single exponential model.

The absorbed dose to the PBL was calculated as a solution of the quadratic Equation (2) for D:

(7) $$D=\frac{\sqrt{\alpha^{2}+4\beta ∆Y(T)G(T)}-\alpha }{2\beta G(T)}.$$

for which 95% confidence interval estimates were calculated using the yield of CAs in PBLs of patients and the computing algorithm recommended in [24].

### 4.5. Statistical and Data Analysis

We used the contingency table method and the chi-squared test to analyze CA spectra expressed as the absolute measured number of CAs. GraphPad Prism 8 software was used for this analysis. Regression analysis was done with the aid of Origin Pro 9 and SigmaPlot 13 software. Algebraic numerical calculations were done using Excel worksheet functions.

## 5. Conclusions

A one-time administration of RAI was safe, as the threshold of 2 Gy for the blood dose was not exceeded. Risk of adverse effects projected from RAI-attributable cytogenetic damage was low, suggesting a good long-term prognosis. Personalized administration would be of special consideration in the event of re-treatments, be it due to recurrent disease or RAI-refractory DTC patients. RAI treatment does not result in persistent accumulation of complex CAs, especially stable ones.

## Figures and Tables

**Table 1:** Biometrical characteristics of DTC patients and radiotherapy treatment data.

Patient No.	Patient’s Biometric Characteristics					Radiotherapy Treatment Data		
	Sex/Age *, y	Weight, kg	Cancer Type	Diagnosis, ICD ^++^	MTS in	EBRT ^+^ Dose, Gy	Cumulative Activity, GBq	Number of RAI Courses
P1	F/50.5	92	folicular	pT4N0M0	lungs	50	40.0	10
P2	F/71.0	87	papilar	pT3aN1aM1	lungs	32; 46	60.4	18
P3	F/40.7	67	refractory	TxN1bM1, pT0N1bM1	lymph nodes, lungs	40	65.4	22
P4	F/64.0	62	folicular	pT4bN1bM1	sternum	90; 27 **	60	16
P5	M/58.0	92	papilar	pT2N1bM1	lymph nodes, lungs	46	46	11
P7	F/69.0	77	refractory	cT0N1M1	lungs, liver	no	80	17
P8	F/55.2	80	papilar	pT3N0 M0	lymph nodes, lungs, mediastinum	no	43	10
P9	F/6.0	16	refractory, papilar	pT3bN1bM1	lymph nodes	no	2.55	3
P10	F/13.7	34	folicular kind of papilar	tumor from the struma ovarii of the right ovary with dissemination along the peritoneum	small pelvis	no	5.6	2
P11	F/75.7	80	folicular	pT3N0M1	lungs	no	44	9

* At the first cytogenetic examination; ^+^ EBRT means External Beam Radiation Therapy; ^++^ ICD means International Classification of Diseases; ** additional EBRT after the 14th RAI course.

**Table 2:** Clinical characteristics of examined patients.

Patient No. (No. of Tests)	No. of RAI Course	Cumulative RAI Activity, GBq	One-Time RAI Activity, GBq	Length of RAI Treatment, yrs	TSH, µUI/mL	Tg, ng/mL	Anti-Tg Ab, UI/mL
P1 (4)	7 of 10	23.0	5.0	3.4	86.83	13.7	9.91
	9 of 10	32.0	4.0	4.4	24.1	17.6	19.18
P2 (2)	19 of 19	56.4	4.0	10.9	88.63	1312	6.5
P3 (2)	22 of 22	63.2	2.2	14.7	44.6	58.3	9.9
P4 (2)	15 of 16	51.5	4.0	7.8	67.12	813.1	3.2
P5 (6)	9 of 11	34.0	4.0	3.9	87.76	94.43	4.8
	10 of 11	38.0	4.0	4.4	N/A	N/A	N/A
	11 of 11	42.0	4.0	5.0	77.4	102.6	<2
P7 (2)	17 of 17	75.9	4.1	13.1	5.88	22.08	<1.2
P8 (2)	8 of 10	28.0	5.0	4.6	>100	74.34	0.15
P9 (5)	1 of 3	0.0	1.0	0	>100	12,730	21.2
	2 of 3	1.0	1.0	0.3	>100	13,456	13.7
	3 of 3	2.0	0.55	0.7	>100	16,033	19.0
P10 (4)	1 of 2	0.0	2.6	0	84.54	467.7	16.27
	2 of 2	2.6	3.0	0.3	81.38	<0.04	15.78
P11 (2)	9 of 9	38.0	6.0	9.0	19.11	12.34	4.0

**Table 3:** All types of CAs in blood lymphocytes of DTC patients detected by mFISH before and after the regular RAI course (types of CAs are the same as in Table 4).

Patient No.	P1				P2		P3		P4		P5					
No. of course	7 of 10		9 of 10		19 of 19		22 of 22		15 of 16		9 of 11		10 of 11		11 of 11	
Days *	0	3	364	367	0	2	0	3	0	2	0	2	182	184	406	408
Cells	240	144	189	420	489	514	580	167	376	252	675	238	490	736	489	339
Ab.cells	35	34	44	111	81	101	110	43	149	127	150	61	100	209	109	92
Stable ab.cells	16	9	23	53	42	62	60	20	90	50	72	28	57	100	47	41
Rec.tr	21	16	29	79	60	79	72	25	132	83	97	45	70	145	61	51
Non.rec.tr	1	6	2	7	14	9	14	8	19	14	29	6	6	30	12	13
Ace	7	8	10	17	11	15	12	8	10	20	18	8	11	33	27	18
Rc	1	1	0	9	0	1	0	2	3	8	3	1	2	6	3	1
Dic	12	12	5	26	5	10	22	3	21	44	28	14	23	32	25	21
Other	2	1	2	3	1	3	0	3	3	3	5	4	2	5	2	2
CCAs	1	3	3	13	11	7	11	1	21	13	14	6	7	26	12	6
Breaks	85	91	96	317	217	241	266	94	455	389	389	170	243	565	286	215
Sum of all CAs	45	47	51	154	102	124	131	50	209	186	194	84	121	277	142	112
**Patient No.**	**P7**		**P8**		**P9**						**P10**				**P11**	
No. of course	17 of 17		8 of 10		1 of 3		2 of 3		3 of 3		1 of 2		2 of 2		9 of 9	
Days *	0	3	0	3	0	3	124	126	259	N/A	0	3	118	121	0	3
Cells	950	421	481	282	860	290	711	703	548		687	525	480	333	400	156
Ab.cells	342	199	68	69	4	18	47	118	66		5	43	32	39	119	54
Stable ab.cells	173	86	34	29	0	3	27	47	35		1	16	13	14	79	27
Rectr	290	145	48	36	0	3	28	49	37		1	16	13	15	99	39
Non.rec.tr	53	35	8	8	0	1	3	9	6		0	2	3	5	12	1
Ace	40	39	12	9	3	6	7	20	9		4	7	10	7	9	15
Rc	2	2	2	3	0	1	0	5	0		0	4	0	3	2	2
Dic	63	59	10	19	1	5	11	30	13		0	10	7	7	14	12
Other	8	3	4	3	0	1	0	5	3		0	3	0	4	0	0
CCAs	59	28	3	3	0	1	1	9	1		0	3	0	1	11	4
Breaks	1095	623	166	157	5	31	94	244	131		6	89	56	78	295	144
Sum of all CAs	515	311	87	81	4	18	50	127	69		5	45	33	42	147	73

* Time from the first cytogenetic examination.

**Table 4:** CAs detected by mFISH after in vitro irradiation of donor blood samples.

NN of Measurements	Dose, Gy	Cells	Ab Cells	Stable Ab Cells	Ace	Rec tr	Non-rec. tr	Dic	Rc	Other *	CCAs	Sum of CAs
5	0	1851	33	11	18	11	2	1	0	1	0	33
4	0.25	1478	61	20	18	21	4	11	2	3	3	62
3	0.5	1012	103	42	18	44	6	30	1	8	3	110
2	0.75	708	99	36	15	40	4	32	5	5	6	107
4	1.0	846	203	61	52	64	17	69	11	8	11	232
3	1.25	843	243	73	52	87	17	92	8	10	20	286
2	1.5	385	136	37	31	53	17	51	10	5	13	180
7	2	1492	844	185	213	352	85	400	35	48	138	1271
4	3	725	611	75	189	326	82	379	34	48	214	1272
5	4	719	675	52	334	380	128	521	45	52	449	1909

* Other—acentric rings, inversions, other incomplete exchanges.

**Table 5:** Complex aberrations in examined patients.

Patient No.	P1				P2		P3		P4		P5					
No. of course	7 of 10		9 of 10		19 of 19		22 of 22		15 of16		9 of 11		10 of 11		11 of 11	
Days *	0	3	364	367	0	2	0	3	0	2	0	2	182	184	406	408
Cells	240	144	189	420	489	514	580	167	376	252	675	238	490	736	489	339
CCAs	1	3	3	13	11	7	11	1	21	13	14	6	7	26	12	6
Stable CCAs	0	2	3	4	1	1	7	0	5	3	4	0	3	7	3	1
% breaks from CCAs	4.5	12	10.4	16.4	21.2	9.5	14.3	4.3	19.5	17	12.3	12.9	10.7	17.3	18.5	9.8
Max. C/B	3/4	3/4	3/3	5/7	5/6	3/4	4/4	3/4	7/12	6/9	4/5	4/5	4/5	5/7	5/9	3/4
% CCAs	2.2	6.4	5.9	8.4	10.8	5.6	2.4	2	10	7	7.2	7.1	5.8	9.4	8.5	5.4
**Patient No.**	**P7**		**P8**		**P9**						**P10**				**P11**	
No. of course	17 of 17		8 of 10		1 of 3		2 of 3		3 of 3		1 of 2		2 of 2		9 of 9	
Days *	0	3	0	3	0	3	124	126	259	N/A	0	3	118	121	0	3
Cells	950	421	481	282	860	290	711	703	548		687	525	480	333	400	156
CCAs	59	28	3	3	0	1	1	9	1		0	3	0	1	11	4
Stable CCAs	9	6	1	0	0	0	0	2	0		0	0	0	0	6	0
% breaks from CCAs	20.6	15.4	6	6.4	0	1	3.1	11.5	3		0	13.5	0	3.8	12.9	14.6
Max. C/A/B	9/11	6/7	4/4	3/4	-	3/3	3/3	3/4	3/4		-	2/5	-	3/3	3/6	5/7
% CCAs	11.5	9	3.4	3.7	-	5.9	2	7.1	1.4		-	6.7	-	2.4	7.5	5.5

* Time from the first cytogenetic examination; % breaks from CCAs means % of breaks from complex aberrations in the total number of breaks; % CCAs means % of complex aberrations in total yield.

**Table 6:** Clonal aberrations in examined patients.

Patient No.	Aberration	Number of Cells in Clone	No. of Sample	Patient No.	Aberration	Number of Cells in Clone	No. of Sample
P5(s1-6)	tr(1;9)(p1;q1)	3(2 + 1)	s1, s4	P1(s1-4)	tr(7;14)(q3;q1)	3(2 + 1)	s1, s4
	tr(1;16)(p2;q1)	3(2 + 1)	s3, s4		tr(7;8)(q1;q23)	2	s4
	tr(7;14)(q3;q1)	3	s1		tr(1;20)(p3;q1) + tr(8;13)(q24;q14)	2	s4
	tr(9;16)(p1;q1)	3(2 + 1)	s3, s4				
	tr 1′-X + tr X′-1-X	2	s3	P2(s1-2)	tr(4;14)(p1;q1) + tr(6;9)(p1;q3).	2	s1
P7(s1-2)	tr(8;10)(p1;q2)	16(10 + 6)	s1, s2				
	tr(3;22)(p2;q1)	8(6 + 2)	s1, s2	P8(s1-2)	tr(2;3)(p1;q1)	2	s1
	tr(7;14)(q3;q1)	4(3 + 1)	s1, s2				
	tr(5;6)(p1;q25)	3(2 + 1)	s1, s2				
	tr(9;18)(p1;q1)	3	s1				
	tr(19;X)(q1;p1)	3	s1	P11(s1-2)	tr(1;4)(q1;p1)	2	s1
	tr(1;7)(p1;q3)	3	s2		tr(6;8)(p1;q2)	2	s1
	tr(X;X)(p1;q1)	3	s1		tr(2;14)(q1;q3)	2	s1
	tr(1;13)(q1;q2)	2	s1				
	tr(3;11)(p1;q1)	2	s1				
	tr(2;9)(p1;p2)	2	s1				

**Table 7:** Aberration yield and cytogenetic dose estimate for examined DTC patients using the frequency of all CAs detected by mFISH.

Patient No. (No. of Course)	$\mathrm{Before}\;Y_{1}\;\pm \;S$E **	T, h	$\mathrm{After}\;Y_{2}\;\pm \;S$E **	∆Y(T) * ± SE **	G(T)	R_0_ mGy/h	Dose, Gy (CI+)
P1 (7)	18.8 ± 2.8	68	32.6 ± 4.8	13.9 ± 5.5	0.251	61.2	0.99 (0.35–1.62)
P1 (9)	27.0 ± 3.8	68	36.7 ± 2.9	9.7 ± 4.8	0.251	45.7	0.74 (0.12–1.34)
P2 (19)	20.9 ± 2.1	68	24.1 ± 2.2	3.3 ± 3.0	0.251	17.3	0.28 (0–0.75)
P3 (22)	22.6 ± 2.0	68	30.0 ± 4.2	7.4 ± 4.7	0.251	35.8	0.58 (0–1.21)
P4 (15)	55.6 ± 3.8	44	73.8 ± 5.4	18.2 ± 6.6	0.272	78.4	1.20 (0.52–1.88)
P5 (9)	28.7 ± 2.1	44	35.3 ± 3.9	6.6 ± 4.4	0.272	34.0	0.52 (0–1.12)
P5 (10)	24.7 ± 2.2	44	37.6 ± 2.3	13.0 ± 3.2	0.272	60.1	0.92 (0.53–1.30)
P5 (11)	29.0 ± 2.4	44	33.0 ± 3.1	4.0 ± 4.0	0.272	22.2	0.34 (0–0.93)
P7 (17)	54.2 ± 2.4	68	73.9 ± 4.1	19.7 ± 4.7	0.251	80.3	1.30 (0.78–1.80)
P8 (8)	18.1 ± 1.9	68	28.7 ± 3.2	10.6 ± 3.7	0.251	49.4	0.80 (0–1.41)
P9 (1)	0.47 ± 0.23	68	6.2 ± 1.5	5.7 ± 1.5	0.251	29.0	0.47 (0.24–0.70)
P9 (2)	7.0 ± 1.0	44	18.1 ± 1.6	11.0 ± 1.9	0.272	52.9	0.81 (0.54–1.07)
P10 (1)	0.73 ± 0.33	68	8.6 ± 1.3	7.8 ± 1.3	0.251	38.3	0.62 (0.40–0.83)
P10 (2)	6.9 ± 1.2	68	12.6 ± 2.0	5.7 ± 2.3	0.251	29.0	0.47 (0.13–0.81)
P11 (9)	36.8 ± 3.0	68	46.8 ± 5.5	10.0 ± 6.3	0.251	47.0	0.76 (0–1.54)
Average	23.53 ± 0.54	60	31.36 ± 0.75	7.83 ± 1.29	0.258	38.1	0.61 (0.31–0.89)

* ∆Y(T) = Y_2_ − Y_1_; ** standard error of the mean; + 95% confidence interval.

## Data Availability

The data presented in this study are available upon request from the corresponding author. The data are not publicly available due to privacy and ethical restrictions.

## Abbreviations

The following abbreviations are used in this manuscript:

mFISH	Multiplex fluorescence in situ hybridization
DTC	Differentiated thyroid cancer
ICD	International Classification of Diseases
RAI	Radioactive iodine
PBLs	Peripheral blood lymphocytes
CAs	Chromosome aberrations
CCAs	Complex chromosome aberrations
Ab.cells	Aberrant cells
Ace	Acentrics
Rc	Centric rings
Dic	Dicentrics
Rec.tr	Reciprocal translocations
Non.rec.tr	Nonreciprocal translocations
EBRT	External beam radiation therapy
Tg	Thyroglobulin
TCH	Thyroid-stimulating hormone
Anti-Tg ABs	Anti-thyroglobulin antibodies
SPM	Secondary primary malignancy
